# How to ensure effective use of media to communicate with healthcare professionals and general public

**DOI:** 10.12669/pjms.345.16172

**Published:** 2018

**Authors:** Shaukat Ali Jawaid, Masood Jawaid

**Affiliations:** 1 Shaukat Ali Jawaid Chief Editor, Pakistan Journal of Medical Sciences, Karachi - Pakistan; 2 Masood Jawaid Associate Editor Pakistan Journal of Medical Sciences, Karachi - Pakistan

**Keywords:** Media, Healthcare Professionals, Healthcare Facilities, Research Scientists, Communication strategy, Health Journalists, Medical Journalists

## Abstract

Image of the medical profession in the public is not so good these days for various reasons. Media, electronic in particular takes pleasure in defaming, humiliating and running malicious campaigns against doctors and healthcare facilities. They run announcement as breaking news and pass sweeping remarks without knowing the full facts of the incidents. Media houses does not employ professionals with some core knowledge on health related issues which they cover. Most of them know nothing about it but they are also reluctant to admit it and pose themselves as experts. Medical profession also shares some blame for all this because they have never organized any orientation courses for health journalists. Yet another reason is the failure of the medical profession to effectively use media to communicate with the healthcare professionals as well as the public. Self-monitoring, accountability, having a good communication strategy, besides other initiatives can go a long way in improving the medical related news coverage thereby enhancing the image of the medical profession as well.

Image of the medical profession in public is not so good these days for various reasons. In fact, the media, both print as well as electronic takes pleasure in not only humiliating and defaming them but also runs malicious campaigns against the healthcare professionals as well as healthcare facilities without any investigations. One of the important reasons for this sad state of affairs is that media houses and their owners, in particular, do not give much importance to health subjects, hence they do not employ professionals who have some basic core knowledge of medicine and health-related issues. They tend to pass sweeping remarks and run breaking news announcements related to any mishap in the health sector putting blame on the doctors and healthcare facilities without getting full details of the events. They also lack the competence to differentiate between medical errors, professional mishaps, near misses and criminal negligence. Most of those covering the health sector does not possess any basic knowledge which is essential but even then they try to pose themselves as experts.

Members of the medical profession are also responsible for this to some extent as neither they nor their professional specialty organizations, healthcare facilities, and other medical institutions have ever thought of taking certain initiatives to organize some orientation courses for the health journalists. Well trained and competent medical journalists is a very rare commodity in Pakistan, medical press too has too many quacks in its ranks. However, as regards the health journalists, less said the better. Though they do not know much but they are also reluctant to admit it.

## Doctor’s interaction with Pharma Industry

Among the many reasons for the present state of affairs is also the doctor’s relationship and interaction with the pharmaceutical trade and industry. The public has a perception that the HCPs are bribed extensively by the industry and they promote their drugs in return for lucrative incentives which includes pleasure trips overseas besides expensive gifts etc. Dr. Chandra Gulhati the Editor of MIMS India is reported to have once said that “Commercial needs of countless fiercely competing pharma companies has led them to depend on the tried and tested 3Cs. Convince if possible, Confuse if necessary and Corrupt if nothing works.” Yet another reason is the failure of the medical profession to use the media effectively to communicate with the public. They need to learn how to create rapport and maintain good relations with Media which matters.

Speaking at the Asia Pacific Association of Medical Editors (APAME) meeting held at Kuala Lumpur Malaysia in 2012 on how to work with media Dr. Tea Shiao Eek had remarked that the medical profession should be prepared to face media accountability. They should either engage the media or risk being ignored. In his presentation he had also discussed various related issues and gave certain suggestions.^1^ In the past, the researchers, as well as healthcare professionals, he had remarked worked in isolation in their laboratories and healthcare facilities. Their work only used to be scrutinized by their colleagues and peers. However, today the situation has changed. Research findings and scientists are thrust into the media limelight. They are accountable to the public who rely on media as a medium of information. With new developments in social media, information as well as misinformation is disseminated at lightning speed. Public reaction can be rapid, overwhelming, irrational and at times even uncontrollable. If the healthcare professionals fail to engage with media, they risk their work being ignored or even misinterpreted.^1^

We need to understand why science matters to public? What is the role of media? What are the reasons for the tension between the scientists and the media? Why to engage and work with the media? A careful analysis will reveal that science matters to the public because now they are more knowledgeable and educated. All latest information is easily accessible. Science affects them, their health, environment, safety, and advancement. Science is responsible for better medicine, more food, and sophisticated gadgets. It is also responsible for global warming, nuclear technology, and genetic engineering. Hence, people wish to know if the research being performed was necessary for humanity. Is it worth all the money that is being spent on it? Will it cause more serious consequences on our bodies or on the environment? How does research improve our lives?

## Tensions between Scientists and Media

Some of the well-known reasons for tension between scientists and the media include that both face certain challenges. The challenges faced by scientists include the distrust- fear of being misquoted. Media gets excited by wild claims which may not be evidence-based and it not only believes but also practices sensationalism. Media also has unrealistic expectations and does not understand the slow process of discovery and the different variables involved in research. Then comes the communication barrier. The scientists and healthcare professionals most often use jargon or abstract approach, technical explanation instead of using easy to understand language. Not only that the scientist, healthcare professionals may not like being questioned and held accountable.

## Roles and responsibilities of media

Media has certain role and responsibilities. Its role includes of being translator, Gatekeeper and acting as a Watchdog. Recognition of these roles will allow the scientists; healthcare professionals anticipate why journalists adopt a certain approach.^1^ However, it is extremely important that media personnel have core knowledge about the subject they are covering. Professional specialty organizations, medical institutions should educate the media personnel by organizing some orientation courses. However, there is a word of caution. Do not give the impression that you wish to educate them because it hurts their ego. It is a fact that apart from a few exceptions, most media personnel do not know much about these subjects but they are also reluctant to admit it.

## Examples

Once we published a manuscript in Pakistan Journal of Medical Sciences on “Medical Errors, causes, consequences, emotional response and resulting behavior change”.^2^ The study concluded that 128 out of 130 residents described some form of error. Serious errors were 19%, minor errors 48%, near misses 19%. Fatigue, long duty hours, inadequate experience, inadequate supervision, complex cases were the common causes. However, 93% became more careful, 86% started taking increased advice from seniors, and 86% started giving more attention to details. However, the electronic media and lay press which covered this study highlighted that the “majority of the doctors make mistakes while treating patients” reports a study published in Pak J Med Sci. They could not appreciate what is actually meant by medical error, what is near misses etc.

Yet in another case, we published a study “Prescription patterns of GPs in Peshawar-Pakistan. Its findings were that essential components of prescription were missing, legibility was poor in 58.5%, Physician name and Registration Number was missing in 89% and 98.2%, 78% had no diagnosis or indication, dosage, duration of use was also not mentioned in 63.8% and 55.4% respectively. Over-prescription of analgesics, antimicrobials, multivitamins, antiulcer drugs were also reported. However, the media again covered it and highlighted that “Majority of doctors in Pakistan prescribe the wrong drugs to their patients”. 3

Media it may be mentioned here tries to simplify scientific findings which are easy to understand for the public. It makes the results relevant to people’s everyday lives. Their challenges include understanding the research process; know how to read scientific papers besides knowing how to link abstract or basic science with daily realities. Working as a Gatekeeper, the media decides what research is worth highlighting and what is newsworthy? However, they also face a challenge to know what is relevant or significant for readers and they also look for Impact Factor. As a Watchdog Media provides analysis, depth and clinical comments besides holding researchers accountable. Here their challenge is how to become familiar with scientific developments; being independent they should not be carried away by claims while they have to be critical of implications of scientific discovery.

## Why work with the Media?

It is essential to ensure that you get the right information across, you are able to control the story. Sensitize the media and public towards science and the process of discovery. To raise awareness and level of debate in society and to gain public feedback on controversial issues. To work with the media, publishing houses maintain Public Relations Departments. Some institutions, universities outsource this to some Public Relations agency or one can also work as one’s own Public Relations Department. It is also essential that one should know the media and its readership, accessibility. One should pitch a story and not a research topic. Take care that you educate the public and desist from promoting yourself. Maintain a good relationship with journalists/Editors and try to establish yourself as a media source.

## Press Conference

While addressing a press conference, prepare three to five key messages on what you wish to say but do not be derailed. For example during a press briefing about a forthcoming conference, if someone puts a question from the media is a strike by the doctors ethical? The response should be it is a very important issue and we will discuss it in detail at a later sitting but today we wish to concentrate on the topic of the press briefing. At times Heads of the State are kept away from the media at a function purposely to ensure that the focus remains on the event. However, one should be prepared to take provocative questions. It is always better to provide some extra reading material to the media personnel. Prepare a press release highlighting the key message which you wish to convey. Be honest, if the research results, findings are inconclusive.

Do not demand to “approve” the story before it goes to print or on air? Don’t be defensive or hostile when questioned about risks, side effects or contradicting opinions. Do not become over exposed to the media positioning yourself as the only expert or try to market your own work. It is better that just one focal person addressed the press conference though there is always a tendency on the part of others present to get some publicity.

## Medical Conferences

Organizers of most of the medical conferences in many countries including Pakistan seldom realize the importance of the media. In some cases these events turn out to be doctor’s social get together with their families with very little scientific contents. The main objective of the organizers remains to collect as much funds as possible from the pharmaceutical trade and industry. Failure to put up a good scientific programme does not attract participants in the scientific sessions and most often these events get very little or no coverage in the press. On the other hand, even small seminar or symposium organized at a small place with few people gets lot of press coverage simply because the organizers had established contact with the media much in advance and they were also provided all the help and facilities. Experienced medical journalists are too busy and they finalize their schedule months in advance for such conferences. If they are taken on board in the very beginning when the conference is being planned so that it does not clash with their other engagements, they are offered help and facilities, it will ensure adequate coverage thereby convey the organizers message even to those who could not attend the conference for various reasons.

## Social Media: Blogs, Twitter, Face Book

One can also use the social media, blogs and Twitter accounts. Digital stories can increase the impact of your research through videos, interactive pictures or diagrams. One can also use virtual collaboration, peer discussions on an informal level. Scientists and Health Care Professionals(HCPs), Healthcare facilities(HCFs) medical institutions should have a communication strategy for engaging the media instead of doing it on ad-hoc and firefighting basis.^1^ HCPs should project their success stories like patients suffering from schizophrenia, epilepsy, and cancer doing some commendable jobs after being treated. One can also engage some teleplay writers. In the 70s, one of the multinational companies marketing an anti-TB drug-financed production of a short telefilm *“Ibrat”* which highlighted the signs and symptoms of tuberculosis, risk factors, the importance of early diagnosis and appropriate treatment, compliance with drug therapy, following the doctor’s advice not to give up medications at their own and failure to do all this leads to relapse and eventual death of the patient. Electro convulsive therapy (ECT) is an effective treatment modality for schizophrenia as well as depression. If you are not using it for certain reasons, do not talk against it, refer the patient to other colleagues who are using it. Talking against ECT to the patients will send a wrong message and it will tarnish the doctor’s image and prestige.

## Some important initiatives

Certain other positive measures which can be taken include promoting the concept of ethical medical practice. National Bioethics Committee formed by Government of Pakistan has finalized Guidelines on Physicians interaction with Pharma Trade and Industry which have also been approved by the PM&DC.^4^,^5^ It needs to be implemented in its letter and spirit. At the same time, the medical profession should start self-monitoring otherwise someone else will do it and then it will be very painful for them. Professional specialty organizations should take a lead in starting monitoring their members and hold them accountable. Similarly the medical institutions, healthcare facilities should be responsible to monitor their members. HCFs should have some media person designated to communicate with the press. They should also put in place some system of complaint registration, undertaking inquiries and investigate the complaints and then also take corrective measures. Findings of such enquiries should also be made public which will go a long way in enhancing the image of the medical profession assuring the public that the profession has put in place some system of accountability. Even in the case of some unpleasant situations, if such a system is in place, it helps a lot to defuse the situation. The press release prepared by these institutions should state the facts and the message in simple, easy to understand language. In research a great deal of press coverage is focused on observational studies in areas where metrics are poorly established, these studies are hypothesis generating. The scientists must remember, there are enormous risks in talking to the lay press. Be careful to ensure that you get the right message across. There are differences in talking to the patient and to the press. While talking to patients the treating physicians try to make sure that they understand but while talking to the press, one has to make every effort to make sure that they do not misunderstand.^6^

Some healthcare professionals are addicted to the lay press, are always keen to get publicity and remain in the news. They know the tricks how to feed the hunger of the journalists and they also understand how to feed them. Journalists can be called at a very short notice to pass on some sensational story or viewpoint. This is certainly neither Research nor news. Hence the healthcare professionals in general and research scientists in particular are advised to desist from such practices.
